# An evaluation of the clinical features of measles virus infection for diagnosis in children within a limited resources setting

**DOI:** 10.1186/s12887-020-1908-6

**Published:** 2020-01-06

**Authors:** Dominicus Husada, Kusdwijono, Dwiyanti Puspitasari, Leny Kartina, Parwati Setiono Basuki, Ismoedijanto

**Affiliations:** Department of Child Health, Faculty of Medicine, Universitas Airlangga / Dr. Soetomo General Academic Hospital, Surabaya, 60286 Indonesia

**Keywords:** Measles infection, Clinical features, Hyperpigmentation, Surabaya Indonesia, Diagnosis, Outbreak, Limited resource setting

## Abstract

**Background:**

Measles is a recurrent health problem in both advanced and developed countries. The World Health Organization (WHO) recommends anti-measles immunoglobulin M (Ig M) as the standard method of detecting the virus; however, many areas still present the inability to perform a serology test of anti-measles IgM. Therefore, a typical clinical feature is necessary to establish the diagnosis of measles. The objective of this study was to evaluate hyperpigmented rash and other clinical features as the diagnostic tools with respect to measles, especially in an outbreak setting.

**Methods:**

In this observational diagnostic study, the inclusion criteria were as follows: between 6 and 144 months of age, fever, maculopapular rash for 3 days or more, accompanied by a cough, or coryza, or conjunctivitis. Those with a prior history of measles vaccination (1–6 weeks) were excluded, in addition to those with histories of corticosteroid for 2 weeks or more and immunocompromised conditions. The samples were taken from Dr. Soetomo General Academic Hospital in Surabaya, Indonesia. We evaluated the sensitivity, specificity, the positive predictive value, and the negative predictive value of such clinical features. Hyperpigmented rash was validated using Kappa and Mc Nemar tests. Anti-measles Ig M was considered as the gold standard.

**Results:**

This study gathered 82 participants. The clinical manifestations of all subjects included fever, cough, coryza, conjunctivitis, Koplik spots, and maculopapular rash (which turns into hyperpigmented rash along the course of the illness). Most maculopapular rashes turn out to be hyperpigmented (89%). Sensitivity, specificity, positive predictive value, and negative predictive values ​​of the combination of fever, maculopapular rash, and hyperpigmented rash were found to be at 90.7, 28.6, 93.2, and 22.2%, respectively. The Mc Nemar and Kappa tests showed *p* values of 0.774 and 0.119, respectively.

**Conclusion:**

The combination of fever, maculopapular rash, and hyperpigmented rash can be used as a screening tool regarding measles infection in an outbreak setting, which can then be confirmed by anti-measles Ig M. Cough, coryza, and Koplik’s spot can be added to this combination, albeit with a slight reduction of sensitivity value.

## Background

Measles is a very contagious and infectious disease [1–5]. The ability of this virus to cause systemic infection, to transmit via aerosols or droplets, and to suppress the response immune till a long time after the infection make measles a serious matter of concern [4, 6]. In 2012–2014, World Health Organization (WHO) estimated that 115,000 people, mostly including children under 5 years of age, die or suffer from sequelae each year as a result of measles [7, 8]. Around 20 million people worldwide are infected by measles every year [9]. Although the prevalence of this disease has decreased more than 90% in Europe, it has still not been completely eliminated and has instead reemerged, with many recent outbreaks in developed countries [2, 3, 9, 10]. Almost every measles infection clinically manifests and can lead to severe and deadly complications, especially for malnourished children in many developing countries around the world [2].

Measles is associated with fever and rashes [3, 9, 11], examples of which are frequently found in daily practice. The causes of fever and rashes are abundant, with most of them resembling each other in terms of clinical symptoms, which may even lead to misdiagnosis [11, 12]. The incubation period with respect to measles is around 7–21 days. Discrete erythematous rashes develop on the patient’s face and neck a day after the disappearance of the Koplik’s spot. Thereafter, the rashes spread throughout the whole body. Typically, they last for 3–7 days. Patients are considered as highly contagious since 4 days before the appearance of the first rash to 4 days after [4, 5, 9, 11, 13, 14]. The maculopapular rash after few days, becomes hyperpigmented. In a few studies, the hyperpigmented rash may be used as a distinctive sign of measles infection [11, 15, 16]. Until today, the published literature regarding the hyperpigmentation of measles has remained very limited.

In general, people with certain skin tones, especially in some areas of Asia and India, are reported to be more susceptible to hyperpigmentation disorder [17]. Melanin, produced by epidermal melanocytes through the enzymatic oxidation of tyrosine, is the primary pigment that determines skin color [17].

Since hyperpigmentation is observed at the final stage of measles, the importance of detecting it for early diagnosis in individual patient is quite limited. Nevertheless, in an outbreak setting, its value becomes much greater as it allows the early identification of cases and the subsequent prevention of further transmission.

Confirmed diagnoses of measles require virus isolation or polymerase chain reaction (PCR) to support clinical judgment. The WHO recommends anti-measles Immunoglobulin M (Ig M) as the standard method to detect measles infection [9, 13, 18]. However, many entities are still incapable of running laboratory test of PCR or anti-measles Ig M, and thus another diagnostic tool is needed in these regards [19–22]. If we can rely on the clinical features of measles, in any stage of the disease, the necessity of providing diagnostic resources would also be reduced significantly. For a country like Indonesia, which has so many remote areas, such a policy can have a huge impact.

Indonesia has been suffering from many outbreaks of vaccine preventable diseases included poliomyelitis, diphtheria, and measles [23–25]. Every year, some measles outbreak cases are found in many places across Indonesia, just like other underdeveloped and developing countries [20–22, 26–31]. . In 2013 and 2014, a total of 11,521 and 12,943 measles cases were reported all over Indonesia [32]. Moreover, the coverage of measles vaccination in some areas in 2013 were lower than 70% [25]. The official report of measles vaccine coverage was 84%, but 700 thousands children may not have even received any measles vaccine [33]. Indonesia performed the measles rubella (MR) vaccine campaign in 2017 (only on Java Island) and 2018 (the rest of the country). In Java Island itself, the coverage reached nearly 100% of the previous target prediction. In the following year, the impact of this campaign was excellent and reduced the prevalence of measles in Java sharply [26]. Unfortunately, in 2018, the coverage outside Java Island was scarce, leaving a big doubt regarding Indonesia’ ability to prevent measles outbreak [26]. In highly endemic countries, additional immunization programs or campaigns are also very important in relation to measkes prevention [2, 21].

The aim of this study was to determine whether the hyperpigmented rashes and other clinical signs and symptoms can be used as sensitive and specific clinical markers of measles infection, which can consequently act as alternative tools to diagnose measles infection without having to check the anti-measles Ig M, especially in an outbreak setting.

## Methods

A diagnostic test was performed with the observational design constructed in the Pediatric Ward and Pediatric Outpatient Clinic, Dr. Soetomo General Academic Hospital, Surabaya. The research samples met the inclusion criteria as follows: children aged 6 to 144 months with fever (at least 38.3^o^ C), with an extensive maculopapular rash for 3 days, accompanied by cough, coryza or conjunctivitis [34]. Maculopapular rashes were categorized as extended if they had already started to spread to the thorax, the abdomen, and the extremities.

The exclusion criteria were as follows: a history of prior measles vaccination (8 days to 6 weeks), a history of previous corticosteroid use (for 2 weeks or more), and immunocompromised conditions (malignancy, immunosuppressive medications, and HIV infection). Previously acquired measles vaccination would raise an individual’s immunoglobulin level such that the IgM will be positive, even at the time when a child is not ill. Such vaccination can also lead to a clinical manifestation of measles, although in a milder form, which is considered one of the adverse events that follow measles immunization [35]. Immunocompromized patients would show somewhat different clinical pictures and entail more complications in their evauation. Further, immunocompromized children also tend to have more complications [4, 35]. The use of corticosteroid for a long time period can make children become immunocompromized.

The minimal sample requirements were identified by the formula below:

$$ {\mathrm{N}}_{\mathrm{total}}=\left\{4.{\left(\mathrm{Z}\upalpha \right)}^2.\uppi .\left(1-\uppi \right)\right\}/{\mathrm{W}}^2\Big\}, $$

in which N = sample size; Z½ α = standard deviation = 1,96 (for α = 0.05); W = precision (0.2); and the value of π (0.85) was taken from previous study [36]. The minimal sample requirement was 49.

The participants of the study submitted their recorded histories and underwent physical examinations. The collected data included the following: age or birth date, address, sex, history of measles vaccination, history of recent disease, and prior illnesses. The nutritional state was determined by CDC 2000 curve, using body weight, length/height, and age. All participants were seen and examined at least twice during the study period. If hospitalized, the participants were followed daily and all of their complications were recorded. Anti-measles Ig M tests were performed at least on the third day after the onset of the rash and were used as the gold standard. These tests used Enzygnost® Anti Measles Indirect ELISA kit (Siemens), with the amount of blood needed for each test being between 3 and 5 ml, and they were conducted within a period of 48 h after the blood drawing. The interpretation of the results were as follows: > 0.3 = positive, < 0.2 = negative, and 0.2- < 0.3 = equivocal. For patients with equivocal results, we conducted the second test on the seventh day after the onset of the first rash. In this second test, the result was considered negative if < 0.2 [37]. WHO recommends that a single serum sample be obtained at the first contact with health personnel, within 28 days after the rash onset. Indeed, IgM ELISA detection is most sensitive 4–28 days after the rash onset [38].

From the data set, our study used seven clinical features (fever, erythematous rash, cough, coryza, conjunctivitis, Koplik’s spot, and hyperpigmented rash) to find the best combination comprising the desired sensitivity, specificity, positive predictive value (PPV), negative predictive value (NPV), and the likelihood ratio, Those clinical features were evaluated repeatedly, and for each combination, we test our dataset separately. The comparison between hyperpigmented rash and anti-measles Ig M was also made by the Kappa and Mc Nemar tests.

Informed consent forms were signed by all the involved parents or guardians. Ethical clearance was obtained from The Research Ethical Committee of Dr. Soetomo General Academic Hospital Surabaya, Indonesia.

## Results

Eighty nine candidates for this study in the beginning, with clinical symptoms of measles according to the CDC criteria (1983), of which 82 samples met the inclusion criteria and provided complete data. Seven participants were excluded: five of them were lost to the follow-up, while out of the remaining two one had a history of steroid intake in the last 2 weeks due to nephrotic syndrome and another had leukemia.

Most participants were in the age group of 12 to less than 72 months. The male group dominated the female group with a ratio of 1.3:1. Among the 82 participants, anti-measles Ig M was found to be positive in 75 children (91.5%). The nutritional statuses of 51 subjects (62.2%) was below normal. Twelve subjects received a single vaccination against measles at the age of 9–12 months, while 11 of them were found to have positive anti-measles Ig M (Table 1). Bronchopneumonia as a complication of measles was found in 18% participants.

**Table 1:** Characteristics of subjects

Characteristics	Total Samples (%) *n* = 82	Anti measles IgM (+) (%) *n* = 75
Sex		
Male	47 (57.3)	44 (58.7)
Female	35 (42.7)	31 (41.3)
Age (mo)		
6 – < 12	19 (23.2)	18 (24.0)
12 – < 72	49 (59.7)	45 (60.0)
72–144	14 (17.1)	12 (16.0)
Nutritional State		
Good Nutrition	31 (37.8)	28 (37.3)
Moderate Malnutrition	44 (53.7)	40 (53.3)
Severe Malnutrition	7 (8.5)	7 (9.3)
Measles Vaccination		
Positive	12 (14.6)	11 (14.7)
Negative	70 (85.4)	64 (85.3)
Vitamin A		
Yes	69 (84.1)	63 (84.0)
No	13 (15.9)	12 (16.0)
Complications		
Bronchopneumonia	15 (18.3)	15 (20.0)
Diarrhea	4 (4.8)	4 (5.3)

The clinical manifestations of all subjects consisted of fever, cough, coryza, conjunctivitis, Koplik’s spots, and maculopapular rash (which becomes hyperpigmented along the course of illness). All participants had a fever and maculopapular rashes, as required in the inclusion criteria, while only 50% had conjunctivitis. Hyperpigmented maculopapular rashes were noted in 89% of all the subjects. All children with Koplik’s spots turned out to have positive anti-measles Ig M (Table 2).

**Table 2:** Clinical manifestations of measles infection

Clinical Manifestations	Total Samples (%) *n* = 82	Anti Measles IgM (+) (%) *n* = 75
Fever	82 (100.0)	75 (100.0)
Cough	73 (89.0)	67 (89.3)
Coryza	70 (85.4)	63 (84.0)
Conjunctivitis	41 (50.0)	35 (46.7)
Maculopapular Rash	82 (100.0)	75 (100.0)
Hyperpigmented Rash	73 (89.0)	68 (90.7)
Koplik’s Spot	32(39.0)	32 (42.7)

The comparison of clinical signs and symptoms between younger (less than 1 year old) and older children is shown in Table 3. Younger children had a lesser prevalence of cough, conjunctivitis, and hyperpigmentation, albeit with more coryzas and Koplik’s spots. Younger children had more complications (6 out of 19, or 31.6%) than the older ones (12 out of 63, or 19.1%).

**Table 3:** Comparison of clinical manifestations of measles infection between younger and older children^a^

Clinical Manifestations	< 1 Year Old		> 1 Year Old	
	Total Samples (%) *n* = 19	Anti measles IgM (+)(%) n = 82	Total Samples (%) *n* = 63	Anti Measles IgM (+) (%) *n* = 57
Fever	19 (100)	18 (100)	63 (100)	57 (100)
Cough	16 (84.2)	15 (83.3)	57 (90.5)	52 (91.2)
Coryza	17 (89.5)	16 (88.9)	53 (84.1)	47 (82.5)
Conjunctivitis	6 (31.6)	5 (27.8)	35 (55.6)	30 (52.6)
Maculopapular Rash	19 (100)	18 (100)	63 (100)	57 (100)
Hyperpigmented Rash	16 (84.2)	15 (83.3)	57 (90.5)	53 (93)
Koplik’s Spot	8 (42.1)	8 (44.4)	24 (38.1)	24 (42.1)

^a^Younger children = age 1 year old or less

The various combinations of the three to six different clinical signs and symptoms involving hyperpigmented rashes showed sensitivity and specificity ranges from 41.3–90.7% and 0–28.6%, respectively (Table 4). The highest sensitivity and specificity values were ​​found in the combination of fever, maculopapular and hyperpigmented rash (90.7 and 28.6%), followed by the combination of fever, rash, cough, coryza, and hyperpigmentation (with 81.3% sensitivity and 28.6% specificity). Both combinations also showed the highest values for PPV, NPV, and positive likelihood ratio (Tables 4 and 5). After adding hyperpigmented rash to the combination of fever, erythematous rash, cough, and coryza or fever, erythematous rash, cough, and Koplik’s spot, the sensitivity, specificity, PPV, NPV, and the likelihood ratios became better. Mc Nemar and Kappa tests on all the combinations of measles’ clinical features were deemed insignificant (Table 5).

**Table 4:** The performance of the clinical features of measles as a diagnostic tool – sensitivity, specificity, and likelihood ratio

Clinical features	Sn (%) (95% CI)	Sp (%) (95% CI)	LLR (%)
F + M + H	90.7 (81.2–95.9)	28.6 (5.1–69.7)	0.175
F + M + B + H	81.3 (70.3–89.1)	28.6 (5.1–69.7)	0.545
F + M + C + H	76.0 (64.5–84.8)	28.6 (5.1–69.7)	0.791
F + M + K + H	41.3 (30.3–50.3)	28.6 (5.1–69.7)	0.124
F + M + B + C + H	66.7 (54.7–76.9)	28.6 (5.1–69.7)	0.545
F + M + B + C + K + H	36.0 (25.5–48.0)	28.6 (5.1–69.7)	0.124
F + M + B	89.3 (79.5–95.0)	14.3 (0.8–58.0)	0.777
F + M + *C*	84.0 (73.3–91.1)	0	0.127
F + M + K	46.7 (35.2–58.5)	14.3 (0.8–58.0)	0.038
F + M + B + *C*	73.3 (61.7–82.6)	14.3 (0.8–58.0)	0.448
F + M + B + *C* + K	41.3 (30.3–53.3)	28.6 (5.1–69.7)	0.124

*Sn* sensitivity, *Sp* specificity, *LLR* likelihood ratio, *F* fever, *M* maculopapular rash, *B* cough, *C* coryza, *K* conjunctivitis, *H* hyperpigmented rash, *CI* confidence interval

**Table 5:** The performance of the clinical features of measles as a diagnostic tool – positive predictive value, negative predicitve value, Mc Nemar and Kappa tests

Clinical features	PPV (%)(95% CI)	NPV (%)(95% CI)	Mc Nemar (p)	Kappa (p)
F + M + H	93.2 (84.1–97.5)	22.2 (3.95–59.8)	0.774	0.119
F + M + B + H	92.4 (82.5–97.2)	12.5 (2.2–39.6)	0.064	0.527
F + M + C + H	91.9 (81.5–97.0)	10.0 (0.2–33.1)	0.011	0.788
F + M + K + H	86.1 (69.7–94.8)	4.3 (0.8–16.1)	< 0.001	0.125
F + M + B + C + H	90.9 (79.3–96.7)	12.5 (1.3–25.8)	0.064	0.527
F + M + B + C + K + H	84.4 (66.5–94.1)	4.3 (0.7–14.9)	< 0.001	0.125
F + M + B	91.8 (82.4–96.7)	11.1 (0.6–49.3)	0.791	0.770
F + M + *C*	90.0 (79.9–95.6)	0	0.359	0.252
F + M + K	85.4 (70.1–93.9)	2.4 (0.1–14.4)	< 0.001	0.048
F + M + B + *C*	90.2 (79.2–95.9)	4.8 (0.2–25.9)	0.009	0.027
F + M + B + *C* + K	86.1 (69.7–94.8)	4.3 (0.8–16.1)	< 0.001	0.125

*PPV* positive predictive value, *NPV* negative predictive value, *F* fever, *M* maculopapular rash, *B* cough, *C* coryza, *K* conjunctivitis, *H* hyperpigmented rash, *CI* confidence interval. McNemar and Kappa tests considered significant only if *p* < 0.05

## Discussion

This study used the clinical features of measles based on CDC criteria (1983) as the inclusion criteria, which consisted of fever, maculopapular rash, and any symptom between cough, coryza, or conjunctivitis [34]. In areas with a high incidence of measles, the use of clinical criteria has a high diagnostic value, which is later confirmed by the positive anti-measles Ig M. These clinical criteria, however, have the opposite effect in areas with low incidence of measles. In this regards, the confirmation of IgM anti-measles using serology test is required [12, 38]. The validity of the clinical criteria is perfect when applied to measles eradication programs in areas with high measles incidence [12, 39] The WHO recommends the application of this clinical criteria (CDC 1983) to serve as the clinical presentation of measles in the measles eradication programme [13, 38, 39]. Several extradermatologic manifestations can also be found in measles cases. Most of such clinical findings regarding measles characterize the disease more cogently than others [40].

Because the signs and symptoms of measles are non-specific and patients with fevers and rashes can try to access any level of medical services, the relevant cases are easily missed [11, 13, 14, 30, 35, 41, 42]. Moreover, several patients showed some less typical features. As identification is always critical, including public health intervention [43], .the differential diagnosis of measles based on the erythematous rashes are many, including infectious diseases like rubella, parvovirus, Group A Streptococcus, adenovirus, non polio enterovirus, erythema infectiosum, infectious mononucleosis, and human herpes virus [5, 11, 14, 42]. Each disease has its own clinical manifestation since its early phase. Thus, it is important to obtain complete information about the history of a disease to make the correct diagnosis [9, 14, 16, 30, 42]. The normal pigmentation of a measles patient’s skin can also be used to predict the hyperpigmentation process. However, this hyperpigmentation feature may not always be seen in Caucasian people [16, 17].

Infectious diseases with hyperpigmentation are rare. Among six “old” diseases with classic exanthems, the first (measles) and second (scarlet fever) diseases can cause hyperpigmentation, but not the third (rubella), fifth (erythema infectiosum), and sixth (exanthema subitum) diseases. The fourth disease – referred to as Staphylococcal Scalded Skin Syndrome – can cause hyperpigmentation, but it involves a very different clinical manifestation compared to measles. Most of the literature regarding post inflammatory hyperpigmentation involves both infectious and non-infectious conditions. The non-infectious diseases are predominant, whereas the infectious diseases are limited [11, 14]. In the literature, postinflammatory hyperpigmentation are mostly caused by any form of inflammation, including infections and allergy-related developments. Skin insults can also result in inflammation and postinflammatory hyperpigmentation [17, 44–46]. The non-infectious causes of hyperpigmentation include drug associated reactions. Some drugs could be involved here, including the antiretro viral drug, interferon alfa, non steroidal anti inflammatory drugs, anti hypertensive, antimalarial drugs, and antibiotics [47–50]. .Some forms of postinflammatory hyperpigmentation are more obvious in people with darker skin colors [17]. Hyperpigmented rashes in measles may follow the same pathway but the data required to determine the same trend is very limited.

In individual setting, the early phase of measles will not show hyperpigmentation, and so this clinical appearance has low value for early diagnoses. However, in an outbreak setting or elimination program, the identification of several early cases is important for the existing community’s benefit. Identifying measles can be possible in some early patients instead of steering the focus towards only one child. Any misclassification of measles case could potentially have considerable impacts, because the patient becomes a source of infection. In an outbreak setting, identification in the initial phases and subsequent prevention of the spreading of the disease is very important. In our setting, even in the outbreak situation, the IgM examination was mostly not available. Clinical manifestations give out important information. The identification of measles in low-prevalence area is more difficult because of the limitation of health workers’ clinical experiences and because many diseases show similar signs and symptoms [12, 13, 39].

The anti-measles Ig M in the appropriate clinical situation ensures the laboratory confirmation of measles [4, 5, 9, 13, 16, 41]. Immunity to measles might not be absolute but depend on the level of preexisting antibodies. The intensity of exposure is also an important factor here [51]. For some clinicians, the distinct morphological features and the distribution of the erythematous rashes, along with a particular cluster of systemic appearances, may give the necessary clues. Careful physical diagnosis entails a complete examination of the rashes and the features of systemic involvement [12]. Hyperpigmentation adds significant value with respect to the identification of measles [4, 5, 11].

In this study, all the patients underwent blood tests 3 days after the onset of the symptoms, and their maculopapular rashes became hyperpigmented and desquamated along the course of their illness. Seventy-five (or 91.5%) out of eighty-two subjects showed positive results with respect to anti-measles IgM. This result corresponds with some previous studies that found 70% positivity of IgM anti-measles when it was performed 3 days after the onset of rashes. The WHO states that 30% of all cases of measles showed negative anti-measles Ig M when the blood tests were performed on the third day since the first symptoms had appeared [38, 52] This result emerged because the anti-measles Ig M has not yet been established at that time. Ig M antibody coincided with the appearance of rashes on the skin of a measles patient. If the blood tests were performed on the third day after the onset of rashes, the Ig M would have been positive in 70% of the cases. Further, when the samples were taken on the seventh day of illness, all cases showed positive anti-measles IgM [38] The sensitivity of anti-measles Ig M can be as high as almost 100% when conducted at least 3 days after the onset of rashes [52, 53]. In developed countries where measles cases have nearly disappeared, the positive predictive value of IgM serology decreases as more false positives are obtained. False positive results can occur because of non specific reactions, the interference of rheumatoid factor, or other infections such as parvovirus B19, Human Herpes Virus (HHV) 6, or rubella [52]. .On the other hand, in low-incidence countries, the NPV would be higher [39]. Anti-measles virus IgM would persist till at least 4 weeks after the rash [52]. In older vaccinated children, measles cases can be associated with the weaning of antibodies. This possibility is quite high especially if children only receive measles vaccination once in their lives. For such children, the dynamics of IgM and IgG should be considered and other diagnostic tests might also be needed [52, 54].

Many participants in this study had nutritional problems. Malnourishment caused immune system disturbances. Indeed, measles infection leads to more problems, both in the nutritional and the immunity aspects. Subjects with measles infections tend to have lower body weights than average children [11, 55]. The measles rashes in malnourished children tend to result in greater confluence and progresses to become dark red in color. Desquamation is marked and occurs in a large scale [4].

This study also showed the difference between younger (less than 1 year old) and older children. A study in Hong Kong by Chan et al. showed a similar comparison in the period between 1999 to 2008. They found that the younger children had modified clinical appearances. This group had shorter duration of fever, earlier appearances of rashes, and fewer incidences of conjunctivitis and hyperpigmentation. There were no differences regarding cough, coryza, Koplik’s spot, and other related compications [56]. Their results are different from ours. Our younger children had more coryza, Koplik’s spot, and other complications, albeit fewer instances of cough, conjunctivitis, and hyperpigmentation, as showed in the Table 3.

A study by Ciccone et al. with 463 participants found the clinical features of measles patients as follows: fever (85%), rash (97%), cough (46%), coryza (48%), and conjunctivitis (17%) [57]. In the study by Chan et al. 165 subjects were recruited, with the following proportions of clinical features: fever (100%), rash (100%), cough (96.4%), coryza (98.25%), conjunctivitis (58.2%), and Koplik’s spot (75.2%) [56]. In our study, all the patients had fever, rash, and at least one of cough, coryza, or conjunctivitis, signs and symptoms which were part of the inclusion criteria.

Although the pathogenesis is not well-known, skin manifestation can be the result of the dissemination of infectious agents from distant sites or via direct inoculation to the cutaneous surface. It also may result from immune or cell mediated responses in the skin [14]. The erythematous rashes in measles patients can be potentially explained by the infection of the dermal endothelial cells and keratinocytes, which are later cleared by the host cells’ immune response [58, 59]. Rashes are manifestations of the measles virus-specific type 1 CD4 and the CD8 T-cell adaptive immune response, along with lymphocyte infiltration into the tissue sites of virus replication, and they coincide with the clearance of the infectious measles virus [54]. The immunocompromised patients often do not develop skin rashes following measles infection [6] Clearance of the virus and the resolution of the rashes are both associated with recovery in almost all children. However, some viral ribonucleic acid (RNAs), like those found in a study in Zambia, persist in multiple locations long after the measles virus is no longer detected [54, 60].

These hyperpigmented rashes is presumably caused by delayed hypersensitivity response against virus antigen [61]. The study by Chan et al. found that hyperpigmented rashes were found in 83% of all the participants [56]. In this study, nine children did not show hyperpigmented rash. This was maybe related with the antibodies received from mothers or vaccinations [61].

Koplik’s spot was considered as a pathognomonic sign of measles virus infection [35, 62, 63]. In this study, the specificity and positive predictive value of Koplik’s spot were both 100%. All patients with Koplik’s spot had positive anti-measles Ig M. This spot was not sensitive because many participants in our study came while rashes had already spread all over their bodies. The Koplik’s spots are usually seen since 1–2 days before until 1–2 days after the onset of the first rash [11, 15, 35, 50]. Other researches found that only 47.4% of the total participants had Koplik’s spots, with a specificity of 86.1% [62]. A review by Perry and Halsey mentioned that Koplik’s spot would only be noticed among 60–70% of measles patients [35]. A study by Zenner et al. in London revealed that the PPV of clinically suspecting measles was 50%, but with Koplik’s spot this PPV would improve to 80%. The sensitivity and specificity in the above study were 62.5 and 86.1%, respectively [62]. In Japan, Kimura et al. showed that the sensitivity and specificity of Koplik’s spots as the diagnostic marker for measles were 48 and 80%, respectively. Out of the 3023 participants in this study,only 717 had Koplik’s spots. The positive rate of these spots in patients with other viruses detected were approximately 20–30%. In conclusion, the Koplik’s spots did not indicate a specific manifestation of measles, although the concomitant specificity related to measles was quite high [64].

In our study, the combination of fever, maculopapular rash, and the hyperpigmented rash had 90.7% sensitivity, 28.6% specificity, 93.2% positive predictive value and 22% negative predictive value. Mc Nemar and Kappa test results for hyperpigmented rashes showed *p* values of 0.774, and 0.119, respectively. The percentages of the clinical features of fever, maculopapular rashes, and hyperpigmented rashes in our study were similar to those found by Chan, especially in subjects under 1 years old. In this study, hyperpigmented rashes occurred in 137 (83%) of all the participants. Conjunctivitis and hyperpigmented rash were deemed as two significant clinical symptoms of measles viral infection [56]. Another study in the United Kingdom by Ramsay et al. found that the combination of fever, cough, coryza, and conjunctivitis were non-discriminatory and were broadly similar for many groups with confirmed infections [42]. On the other hand, no specific clinical features were consistently associated with Group A Streptococcus (GAS), parvovirus B19, or even HHV 6 infections [42]. Most scientific articles related to hyperpigmentation described the postinflammatory hyperpigmentation, albeit within a very limited portion of infectious diseases [17]. In non-infectious diseases, hyperpigmentation could be found in relation to many conditions such as acne, atopic dermatitis, psoriasis, impetigo, lichen planus, pytiriasis rosea, allergic and photo-contact, drug reactrion, effect of laser therapy, and insect bites [17].

In their study, Hutchins et al. reported the sensitivities and specificities of four studies using the same clinical variables that were used in our research (fever, rashes, and at least one of cough, coryza, and conjunctivitis; serological confirmation by IgM or enzyme immunoassays (EIA)). In Florida, California, and New York in the 1980s (182 participants), the sensitivity, specificity, the PPV, and the NPV of the clinical case definition were 88, 48, 74, and 70%, respectively. In New York between 1994 and 1995 (99 participants, between 1 and 14 years old) and in Venezuela between 1993 and 1995 (379 participants, between 1 and 14 years old), the sensitivity, specificity, PPV, and NPV values were 50, 69, 4, and 98%, and 76, 51, 35, and 86%, respectively. The fourth study in Suriname (121 participants, 52% of who were < 5 years old, and 15% were 15 years old or older) showed the sensitivity, specificity, PPV, and NPV values of 100, 23, 1, and 100%, respectively. The sensitivity values of those studies was somewhat similar or lower than our results, while the specificity values were higher than ours. All four studies considered different incidences of measles. The low incidence of measles churned out low specificity and PPV values [39].

In our study, the inclusion criteria included fever which limits the possibility of non-infectious diseases, although several allergic and immunological diseases could also entail fever [65]. The high sensitivity value (90.7%) for fever, maculopapular rashes, and hyperpigmented rashes in our study indicates that these parameters can be used for screening with respect to measles surveillance. However, a diagnostic tool is valid only for the Mc Nemar test with *p* ≥ 0.05 and for Kappa values ​​with *p* < 0.05. Therefore the combination of fever, maculopapular rashes, and hyperpigmented rashes (combined with either cough, coryza, conjunctivitis, and Koplik’s spot) could not be used as a replacement of the anti-measles IgM value.

In practice, when we found suspicious cases of measles, and became surer about our diagnosis by evaluating the hyperpigmentation of our patients, we could follow the outbreak protocol in our community. Indeed, we do not have to wait for the IgM examination to be available. However, if it is available, we can confirm the clinical findings with the IgM examination itself and use it to prevent the further spread of the disease. Once again, the benefit of this hyperpigmentation is more at epidemiological level.

To our knowledge, the study of clinical manifestation focusing on hyperpigmentation in measles disease remain very limited in the existing literature [11, 16, 56, 66]. There may be variations in clinical appearances among patients in many countries. This study can provide be a base upon which the Ministry of Health in Indonesia can modify its diagnosis criteria by including hyperpigmentation, while the initial erythematous rash disappears, as one of the screening tools to diagnose measles within an outbreak setting. Lower predictive values emphasise the importance of the local contexts of the patients and the epidemiological variations, as described in other studes about Koplik’s spots [62].

This study was limited to a single-center. Most of our children were Javanese in ethnicity and had the skin type IV-V (Fitzpatrick skin types IV-V). Children with skin type VI can only found in the eastern part of Indonesia and not included in our study. Hyperpigmentation is rarer in Caucasian people. In general, people with darker skin tend to have more common hyperpigmentation [16, 17, 67]. The other limitation was related to the usage of PCR: we did not use PCR as the gold standard, since the WHO recommendations accept the anti-measles IgM as well. We also did not examine other causes of the disease (with maculopapular rashes) in this study. Some definitive laboratory examinations were not available in our country.

At the moment, measles is included in a short list of potentially eradicable disease [2, 13]. As the developing country with low coverage of vaccination and high incidence of measles, Indonesia is still likely witness more outbreaks in the future. The higher vaccination coverage is the key factor that can tackle this problem, as seen in many other countries [2, 13, 20–22, 31, 68]. The ability to diagnose based on clinical appearances in most of the measles cases is imperative. In addition, rapid and prompt feedback to public health officers is crucial for timely interventions [14, 22, 52]. The outbreak setting is certainly different from the individual patient approach within the clinical settings.

## Conclusions

Hyperpigmented rash was noted in most subjects with measles in this study. Combined symptoms of fever, maculopapular rashes, and hyperpigmented rashes can be used as a screening tool to detect measles infection, even before the confirmatory serology test of anti-measles Ig M takes place. Nevertheles, the former cannot replace the serologic test of anti-measles Ig M. The benefits of the former, hoever, gain more importance in an outbreak setting, where a clinician should make definitive diagnoses of early cases as soon as possible. In most individual situations, clinicians should rely on the clinical features other than hyperpigmentation as the initial working tool, since hyperpigmentation was found at a later stage of the disease.

## Supplementary information

**Additional file 1.** The Presence of Clinical Manifestations in Every Participant.

## Data Availability

The raw data of this study are available as Additional file.

## Abbreviations

CD4: Cluster of differentiation 4
CD8: Cluster of differentiation 8
CDC: Center for disease control and prevention
EIA: Enzyme immunoassay
GAS: Group A *Streptococcus*
HHV: Human herpesvirus
IgG: Immunoglobulin G
IgM: Immunoglobulin M
MR: Measles rubella
NPV: Negative predictive value
PCR: Polymerase chain reaction
PPV: Positive predictive value
WHO: World Health Organization
